# Bilobalide protects against ischemia/reperfusion-induced oxidative stress and inflammatory responses via the MAPK/NF-휅B pathways in rats

**DOI:** 10.1186/s12891-020-03479-9

**Published:** 2020-07-09

**Authors:** Ying Li, Jiliang Jiang, Liangcheng Tong, Tingting Gao, Lei Bai, Qing Xue, Jianxin Xing, Qin Wang, Haoran Lyu, Min Cai, Zhongyang Sun

**Affiliations:** 1 Department of Orthopedics, Air Force Hospital of Eastern Theater, Anhui Medical University, Nanjing, China; 2 Department of Neurosurgery, Yulin First Hospital, the Second Affiliated Hospital of Yan’an University, Yulin, China; 3 Department of Orthopedics, Yuhuatai Hospital, Nanjing, China; 4 Department of Orthopedics, Zhangwenxin Hospital, Nanjing, China

**Keywords:** Bilobalide, Skeletal muscle ischemia/reperfusion injury, Oxidative stress, Inflammatory responses, MAPK/NF-휅B pathways

## Abstract

**Background:**

Clinically, skeletal muscle ischemia/reperfusion injury is a life-threatening syndrome that is often caused by skeletal muscle damage and is characterized by oxidative stress and inflammatory responses. Bilobalide has been found to have antioxidative and anti-inflammatory effects. However, it is unclear whether bilobalide can protect skeletal muscle from ischemia/reperfusion injury.

**Methods:**

The effects of bilobalide on ischemia/reperfusion-injured skeletal muscle were investigated by performing hematoxylin and eosin staining and assessing the wet weight/dry weight ratio of muscle tissue. Then, we measured lipid peroxidation, antioxidant activity and inflammatory cytokine levels. Moreover, Western blotting was conducted to examine the protein levels of MAPK/NF-휅B pathway members.

**Results:**

Bilobalide treatment could protected hind limb skeletal muscle from ischemia/reperfusion injury by alleviating oxidative stress and inflammatory responses via the MAPK/NF-휅B pathways.

**Conclusions:**

Bilobalide may be a promising drug for I/R-injured muscle tissue. However, the specific mechanisms for the protective effects still need further study.

## Background

Limb ischemia/reperfusion (I/R) injury is a life-threatening syndrome that is often caused by trauma, primary thrombosis, arterial embolism, limb or flap reattachment, artery transplantation, prolonged tourniquet application, and abdominal compartment syndrome [1]. Mild I/R injury can lead to skeletal muscle fibrosis, persistent damage and necrosis, which can affect limb function; in severe cases, patients may require amputation. Patients with severe limb I/R injury can develop multiple organ dysfunction syndromes that threaten their lives [2].

Though the mechanisms of skeletal muscle I/R injury are diverse, a growing body of evidence has proved that oxidative stress and inflammatory responses have important roles in the progress of skeletal muscle I/R injury [3, 4]. Overproduction of reactive oxygen species (ROS) has been observed in I/R-injured organs. Excessive production of ROS can initiate lipid peroxidation, inactivate antioxidative stress-related proteins, and aggravate I/R injury [4]. Thus, injury can stimulate lipid peroxidation of biological membranes. Notably, nicotinamide adenine dinucleotide phosphate (NADPH) oxidase activity and the levels of F_2_-isoprostanes and malondialdehyde (MDA) are often used as indicators of oxidative stress [3–6]. Various defense mechanisms are induced by ROS-induced injury, and the levels of antioxidants such as catalase (CAT), glutathione peroxidase (GSH-Px) and superoxide dismutase (SOD) are often used as indicators of oxidative stress [3, 7]. In addition, ROS-induced injury can promote the formation and release of many inflammatory cytokines, such as tumor necrosis factor alpha (TNF-α), interleukin 6 (IL-6) and interleukin 1 beta (IL-1β) [4–9]. Under oxidative stress or inflammatory conditions, the nuclear factor kappa-B (NF-휅B) and mitogen-activated protein kinase (MAPK) pathways can be activated [10]. Many I/R injury models have been reported to elicit activation of the p38, JNK, ERK1/2 and p65 pathways [8, 9]. Specifically, it has been reported that activation of p38 and ERK1/2 is involved in renal I/R injury [11]. Furthermore, blockade of P38 α and β may protect the lungs from acute I/R injury by reducing the expression of IL-1β [12]. Moreover, 6-gingerol exerts protective effects against I/R-induced intestinal mucosal injury by inhibiting the formation of ROS and the activation of p38 and NF-κB [13]. It has also been reported that gypenoside protects cardiomyocytes against I/R injury through inhibition of MAPK pathway signaling and NF-κB p65 translocation into nuclei [14].

Based on this information, inhibition of the production and release of ROS and inflammatory cytokines is considered one of the strategies for addressing limb I/R injury. *Ginkgo biloba* can alleviate injury associated with stroke or myocardial infarction through its powerful antioxidant and anti-inflammatory properties [15, 16]. Bilobalide is one of the major pharmacological components of *Ginkgo biloba*. It has been reported that bilobalide can protect neurons and endothelial cells from oxidative and inflammatory stress injury [15, 16]. However, at present, little is known about whether bilobalide can protect skeletal muscle from I/R injury. In this study, we hypothesized that bilobalide could alleviate skeletal muscle damage caused by I/R injury by relieving oxidative stress and systemic inflammatory responses. To test this hypothesis, we examined the effects of bilobalide on skeletal muscle using methods including hematoxylin and eosin (H&E) staining and assessment of the wet weight/dry weight ratio of muscle tissue. Then, we measured lipid peroxidation, antioxidant activity, and inflammatory cytokine levels using test kits. Finally, we examined the activation of members of the MAPK and p65 NF- κB pathways via Western blotting.

## Methods

### Animals and ethics statement

For this study, male Sprague-Dawley rats aged 6–8 weeks were obtained from the Jiangsu Province Laboratory Animal Center. All rats were sacrificed by cervical dislocation after the experiment. All experimental procedures were approved by the Ethics Committee for Animal Use of Anhui Medical University.

### Rat model of femoral artery I/R

All rats were treated with 3 h of ischemia and 24 h of reperfusion. The femoral artery was found, and the blood supply was interrupted with an atraumatic microvascular clamp. To completely block the blood flow of the hind limbs, a band was fitted around the right greater trochanter after limb exsanguination. The blood supply was restored after 3 h (to stop the ischemic conditions) by removing the clamp and band. Blood was allowed to reperfuse the area for an additional 24 h before sampling [8, 9].

### Experimental groups and drug treatment

Thirty-two rats were randomly assigned to the following four groups of 8 rats each: (1) the sham group, (2) the I/R group (3) the I/R + bilobalide-low (4 mg/kg) group, and (4) the I/R + bilobalide-high group (12 mg/kg).

Bilobalide was purchased from Shanghai Winherb Medical Development. In the sham group, the femoral artery was only isolated for 3 h, and the rats were injected with saline intraperitoneally before reperfusion. In the I/R group, the femoral artery was blocked for 3 h, and the rats were injected with saline intraperitoneally before reperfusion. In the I/R + bilobalide-low group, the femoral artery was blocked for 3 h, and the rats were intraperitoneally injected with saline containing 4 mg/kg bilobalide before reperfusion. In the I/R + bilobalide-high group, the femoral artery was blocked for 3 h, and the rats were intraperitoneally injected with saline containing 12 mg/kg bilobalide before reperfusion [7]. In most studies, 3–15 mg/kg bilobalide has been used as the effective concentration for experiments [17–19]. Therefore, we chose 4 mg/kg and 12 mg/kg in this study to further explore the effects of bilobalide on skeletal muscle I/R injury. Notably, 12 mg/kg bilobalide had no effects on the skeletal muscles of healthy rats (Supplementary Figure 1).

### Morphometric analysis

Gastrocnemius muscle tissues were embedded in paraffin and cut into 3–5 μm-thick sections. For histological quantification of hind limb muscle fiber injury, five random fields were evaluated for damage. Uninjured fibers were characterized as having well-defined borders and morphologic uniformity without holes or breaks, while injured fibers exhibited broken, fragmented fiber morphologies. Blinded observers scored the morphological impairment according to previously published methods based on muscle fiber disorganization and degeneration and on inflammatory cell infiltration. A score of 0 indicated no damage, 1 indicated mild damage, 2 indicated moderate damage, 3 indicated severe damage, and 4 indicated very severe damage. The damage score was calculated as the sum of each of the parameters [4, 8, 20].

### Wet weight/dry weight ratio of muscle tissue

The tibialis anterior muscle of each rat was weighed immediately after it was taken from the right hind limb (wet weight). The samples were dehydrated and weighed again (dry weight). The level of tissue edema was evaluated by the wet/dry ratio as follows: wet/dry ratio = (wet weight/dry weight) × 100% [4, 8].

### Measurement of lipid peroxidation and antioxidant activity

NADPH oxidase activity and F_2_-isoprostane levels in muscles were determined as described previously [5, 6]. MDA concentrations were assessed by using a commercial kit (A003–1-2, Nanjing Jiancheng Biotechnology Institute, China). The activity levels of SOD (A001–3-2), GSH-Px (A005–1-2) and CAT (A007–1-1) in muscle homogenate were measured using kits (Nanjing Jiancheng Biotechnology Institute, China) according to the manufacturer’s instructions [4].

### Measurement of myeloperoxidase (MPO) activity and inflammatory cytokine levels

An MPO Detection Kit (A044–1-1, Nanjing Jiancheng Biotechnology Institute, China) was used to determine the activity of MPO by measuring the H_2_O_2_-dependent oxidation of 3,3′-dimethoxybenzidine in order to assess neutrophil infiltration.

The levels of TNF-α (H052), IL-6 (H007) and IL-1β (H002) in serum were measured with ELISA kits (Nanjing Jiancheng Biotechnology Institute, China) according to the manufacturer’s instructions [4, 8].

### Western blotting

Muscle tissues were lysed with RIPA buffer (Thermo Fisher Scientific, USA). Protein samples were boiled with loading buffer and then separated by SDS/PAGE prior to being transferred to a nitrocellulose membrane. The nitrocellulose membrane was incubated for 5 h at room temperature with primary antibodies. The primary antibodies included anti-P-P38 (ab4822; 1:1000 dilution; Abcam, USA), anti-P38 (ab31828; 1:1000 dilution; Abcam, USA), anti-P-ERK1/2 (ab223500; 1:400 dilution; Abcam, USA), anti-ERK1/2 (ab17942; 1:1000 dilution; Abcam, USA), anti-P-JNK (ab227061; 1:1000 dilution; Abcam, USA), anti-JNK (ab225572; 1:1000 dilution; Abcam, USA), anti-P65 (ab16502; 1:1000 dilution; Abcam, USA) and anti-β-actin (ab8227; 1:2000 dilution; Abcam, USA). The blots were incubated with a secondary antibody (1:10,000; Jackson, USA), and the secondary antibody was detected by using Tanon imaging software [4].

### Statistical analysis

Data are presented as the means ± SEs. Statistical differences among groups were analyzed by one-way analysis of variance (ANOVA) with a Bonferroni post hoc test to determine group differences in all numerical data. All statistical analyses were performed with SPSS software version 19.0. *P* < 0.05 was considered statistically significant.

## Results

### Bilobalide attenuates skeletal muscle damage caused by I/R injury

**Fig. 1:**
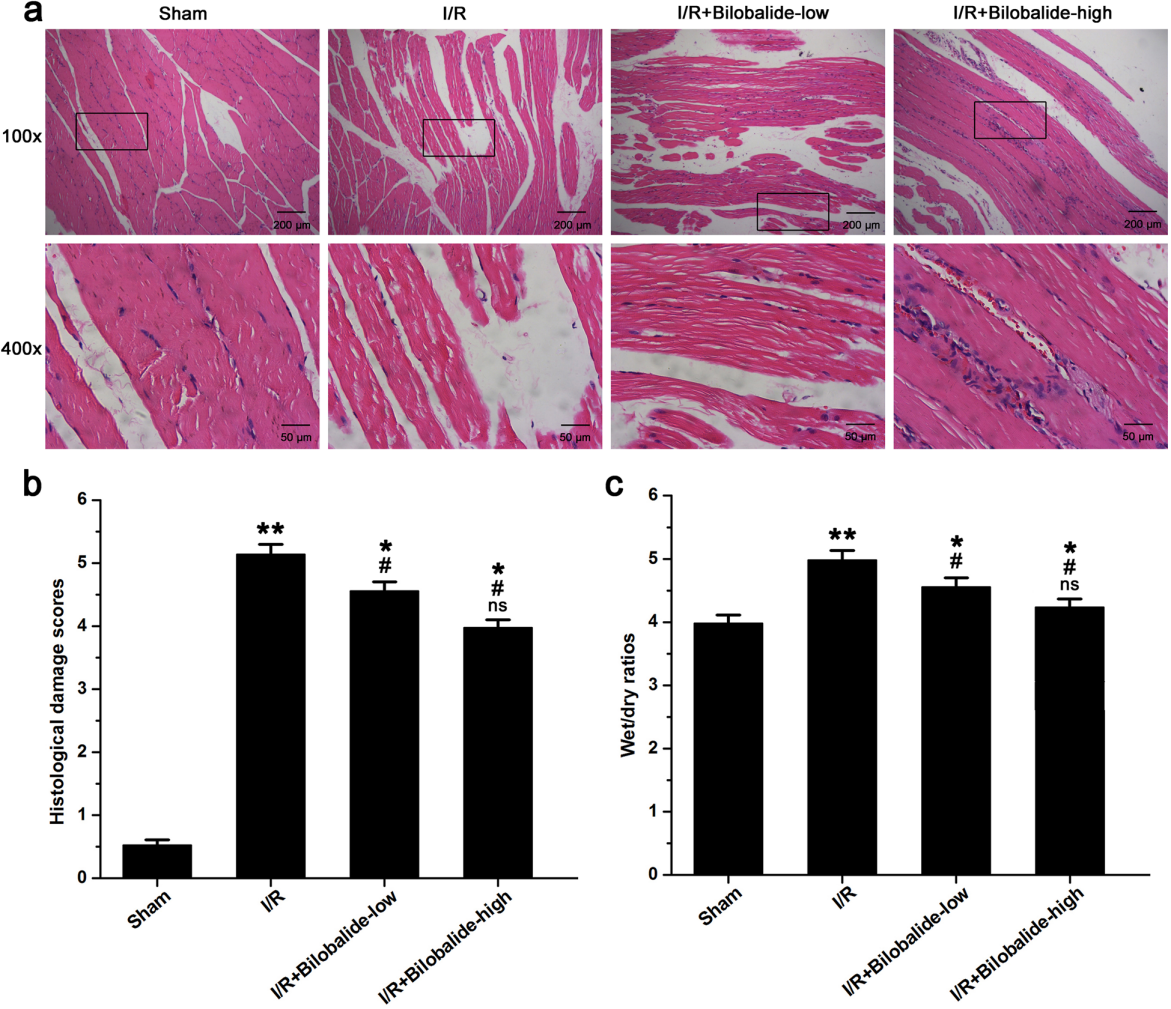
Bilobalide ameliorated skeletal muscle damage and edema induced by I/R. **a** Representative photomicrographs of skeletal muscle tissue following H&E staining (upper panel magnification = 100×, scale bars = 200 μm; lower panel magnification = 400×, scale bars = 50 μm). Uninjured fibers were characterized as having well-defined borders and morphologic uniformity without holes or breaks, while injured fibers exhibited broken, fragmented fiber morphologies. **b** Quantification of histological damage scores in the different groups. For histological quantification of hind limb muscle fiber injury, five random fields were evaluated for damage. The histological damage scores were based on muscle fiber disorganization and degeneration and on inflammatory cell infiltration. **c** Edema is presented as the wet weight/dry weight ratios of skeletal muscle in the different groups. ^*^P < 0.05 and ^**^p < 0.01 compared with the Sham group; ^#^*p* < 0.05 compared with the I/R group; ns = not significant compared with the I/R + bilobalide-low group. *n* = 8 per group

Muscular tissue injury was evaluated by the H&E staining. Uninjured fibers were characterized as having well-defined borders and morphologic uniformity without holes or breaks, while injured fibers exhibited broken, fragmented fiber morphologies. Histological damage scores were assigned based on muscle fiber disorganization and degeneration and on inflammatory cell infiltration. Muscle fiber injury, neutrophil infiltration, sarcoplasm dissolution and erythrocyte diapedesis were observed in the I/R group animals but not in those of the sham group animals (Fig. 1a). Bilobalide treatment alleviated the degree of muscle injury (Fig. 1a). Consequently, the histological damage scores of the I/R group rats were higher than those of the sham group rats (Fig. 1b, *p* < 0.05). Bilobalide decreased histological damage scores in muscle tissue following I/R injury (Fig. 1b, *p* < 0.05). However, the histological damage scores were higher in the I/R + bilobalide-low group and the I/R + bilobalide-high group than in the sham group (Fig. 1b, *p* < 0.05). Moreover, the histological damage scores tended to be lower in the I/R + bilobalide-high group than in the I/R + bilobalide-low group, but the difference between groups was not statistically significant (Fig. 1b, *p* > 0.05).

In addition, skeletal muscle wet/dry ratios were determined to detect changes in tissue edema. The skeletal muscle wet/dry ratio in the I/R injury group was higher than that in the sham group (Fig. 1c, *p* < 0.05). The wet/dry ratios of the animals in the I/R injury groups treated with bilobalide were lower than those of the animals in the untreated I/R injury group (Fig. 1c, *p* < 0.05). However, the wet/dry ratios were higher in the I/R + bilobalide-low group and the I/R + bilobalide-high group than in the sham group (Fig. 1c, *p* < 0.05). Moreover, the wet/dry ratios tended to be lower in the I/R + bilobalide-high group than in the I/R + bilobalide-low group, but the difference between the groups was not statistically significant (Fig. 1c, *p* > 0.05).

### Bilobalide alleviates local oxidative stress in the skeletal muscles of rats with I/R injury

**Fig. 2:**
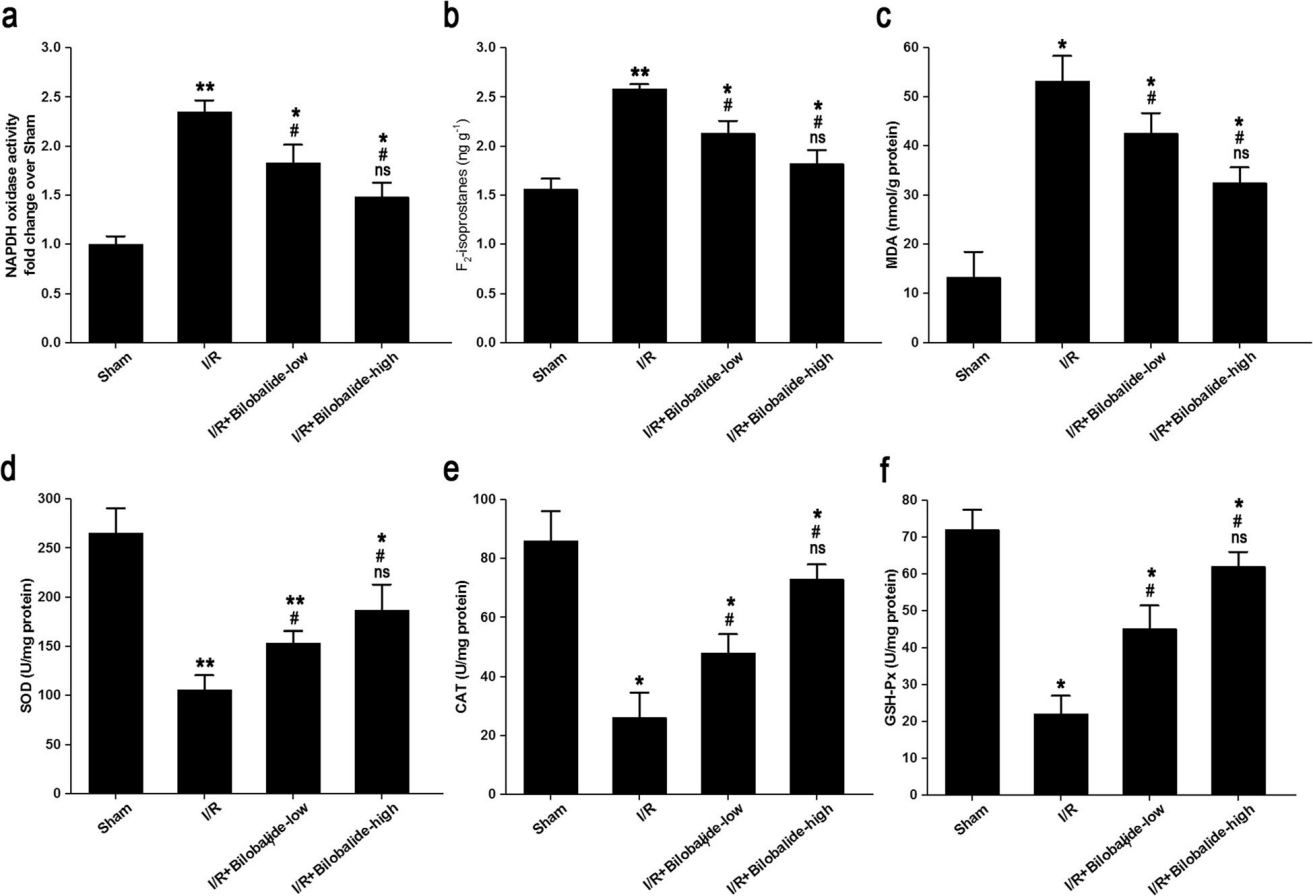
Bilobalide reduced NADPH oxidase activity and F_2_-isoprostane and MDA levels and enhanced SOD, CAT, and GSH-Px activity in skeletal muscle following I/R. The activity of NADPH oxidase (**a**) and the levels of F_2_-isoprostanes (**b**) and MDA (**c**) were increased in muscle tissue after I/R injury, while the activity of SOD (**d**), CAT (**e**), and GSH-Px (**f**) was decreased. Bilobalide treatment reversed the injury-induced changes. ^*^P < 0.05 and ^**^p < 0.01 compared with the sham group; ^#^*p* < 0.05 compared with the I/R group; ns = not significant compared with the I/R + bilobalide-low group. *n* = 8 per group

NADPH oxidase activity (Fig. 2a, *p* < 0.05), F_2_-isoprostane levels (Fig. 2b, *p* < 0.05) and MDA levels (Fig. 2c, *p* < 0.05) were higher in the I/R group than in the sham group. Additionally, the tissue NADPH oxidase activity and F_2_-isoprostane and MDA levels were lower in the bilobalide-treated I/R groups than in the I/R group (Fig. 2a-c, *p* < 0.05). However, the NADPH oxidase activity and F_2_-isoprostane and MDA levels in the I/R + bilobalide-low group and the I/R + bilobalide-high group were higher than those in the sham group (Fig. 2a-c, *p* < 0.05). Moreover, the values of these parameters tended to be lower in the I/R + bilobalide-high group than in the I/R + bilobalide-low group, but the differences between the groups were not statistically significant (Fig. 2a-c, *p* > 0.05).

SOD (Fig. 2d, *p* < 0.05), CAT (Fig. 2e, *p* < 0.05) and GSH-Px (Fig. 2f, *p* < 0.05) activity levels were significantly lower in the I/R group than in the sham group. Additionally, the tissue SOD, CAT and GSH-Px activity levels were higher in the bilobalide-treated I/R groups than in the I/R group (Fig. 2d-f, *p* < 0.05). However, the activity of these enzymes was lower in the I/R + bilobalide-low group and the I/R + bilobalide-high group than in the sham group (Fig. 2d-f, *p* < 0.05). Moreover, the tissue SOD, CAT and GSH-Px activity levels tended to be higher in the I/R + bilobalide-high group than in the I/R + bilobalide-low group, but the differences between the groups were not statistically significant (Fig. 2d-f, *p* < 0.05).

### Bilobalide ameliorates the skeletal muscle inflammatory response in rats with I/R injury

**Fig. 3:**
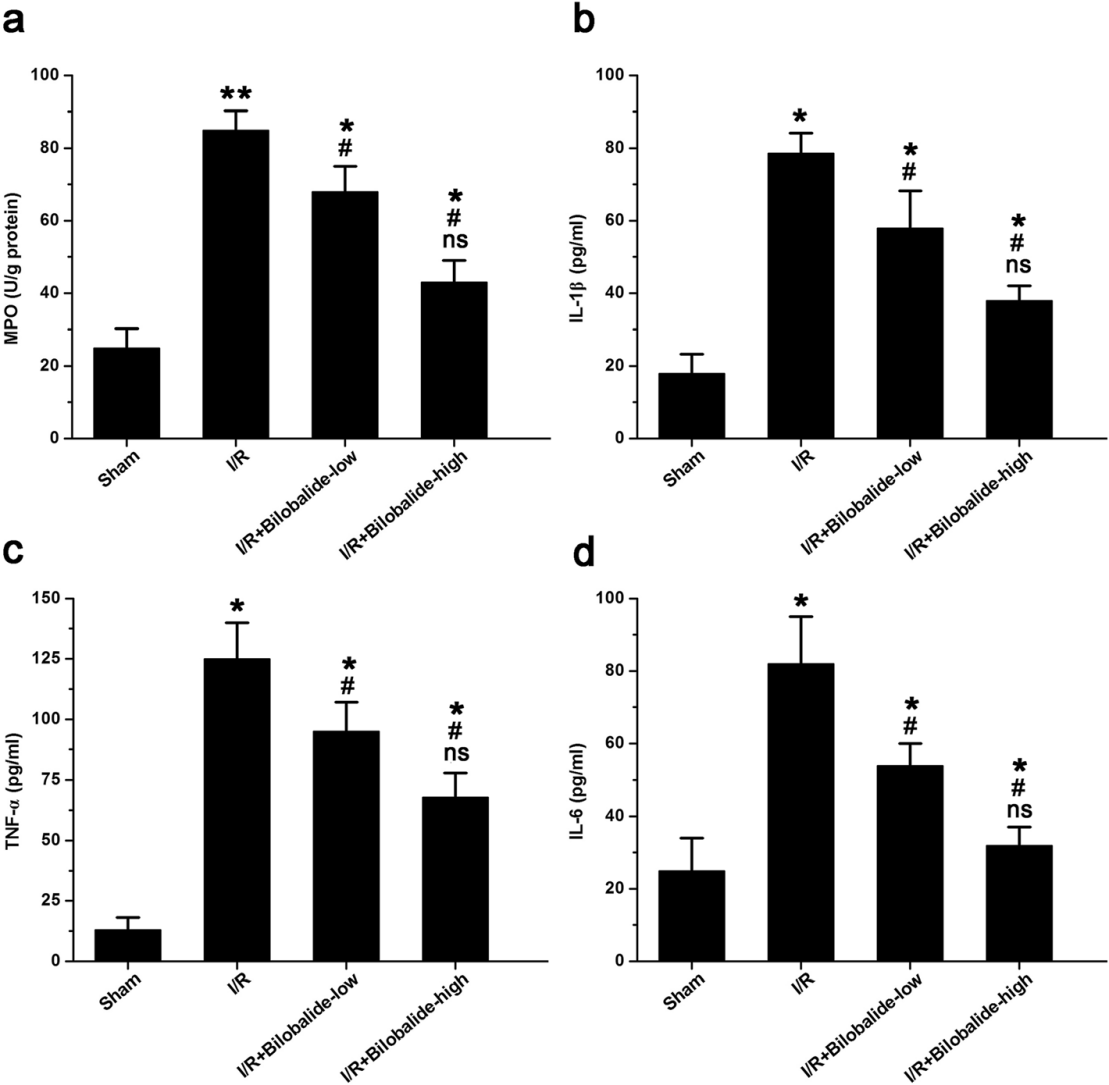
Bilobalide relieved the skeletal muscle inflammatory response post I/R. The activity of MPO (**a**) and the levels of IL-1β (**b**), TNF-α (**c**), and IL-6 (**d**) were increased in muscle tissue after I/R injury, but bilobalide significantly alleviated these injury-induced responses. ^*^P < 0.05 and ^**^p < 0.01 compared with the sham group; ^#^*p* < 0.05 compared with the I/R group; ns = not significant compared with the I/R + bilobalide-low group. *n* = 8 per group

The activity of MPO in the I/R group was higher than that in the sham group (Fig. 3a, *p* < 0.05), which indicated the occurrence of neutrophil infiltration and inflammatory cytokine activation after I/R. Treatment with bilobalide markedly attenuated the I/R-induced increase in MPO activity (Fig. 3a, *p* < 0.05). However, MPO activity was higher in the I/R + bilobalide-low group and the I/R + bilobalide-high group than in the sham group (Fig. 3a, *p* < 0.05). Moreover, MPO activity tended to be lower in the I/R + bilobalide-high group than in the I/R + bilobalide-low group, but the difference between the groups was not statistically significant (Fig. 3a, *p* > 0.05).

Additionally, the levels of IL-1β (Fig. 3b, *p* < 0.05), TNF-α (Fig. 3c, *p* < 0.05) and IL-6 (Fig. 3d, *p* < 0.05) were significantly higher in the I/R group than in the sham group. Similar to the effects on MPO activity, bilobalide treatment attenuated the I/R-induced increases in inflammatory cytokines levels; the levels of IL-1β (Fig. 3b, *p* < 0.05), TNF-α (Fig. 3c, *p* < 0.05) and IL-6 (Fig. 3d, *p* < 0.05) in the bilobalide-treated I/R groups were significantly lower than the levels in the I/R group. However, the levels of IL-1β (Fig. 3b, *p* < 0.05), TNF-α (Fig. 3c, *p* < 0.05) and IL-6 (Fig. 3d, *p* < 0.05) were higher in the I/R + bilobalide-low group and the I/R + bilobalide-high group than in the sham group (Fig. 3a, *p* < 0.05). Moreover, the levels of IL-1β (Fig. 3b, *p* < 0.05), TNF-α (Fig. 3c, *p* < 0.05) and IL-6 (Fig. 3d, *p* < 0.05) were lower in the I/R + bilobalide-high group than in the I/R + bilobalide-low group, but the differences between the groups were not statistically significant (Fig. 3a, *p* > 0.05).

### Bilobalide suppresses activation of the p38, ERK1/2, JNK, and NF-κB p65 pathways

**Fig. 4:**
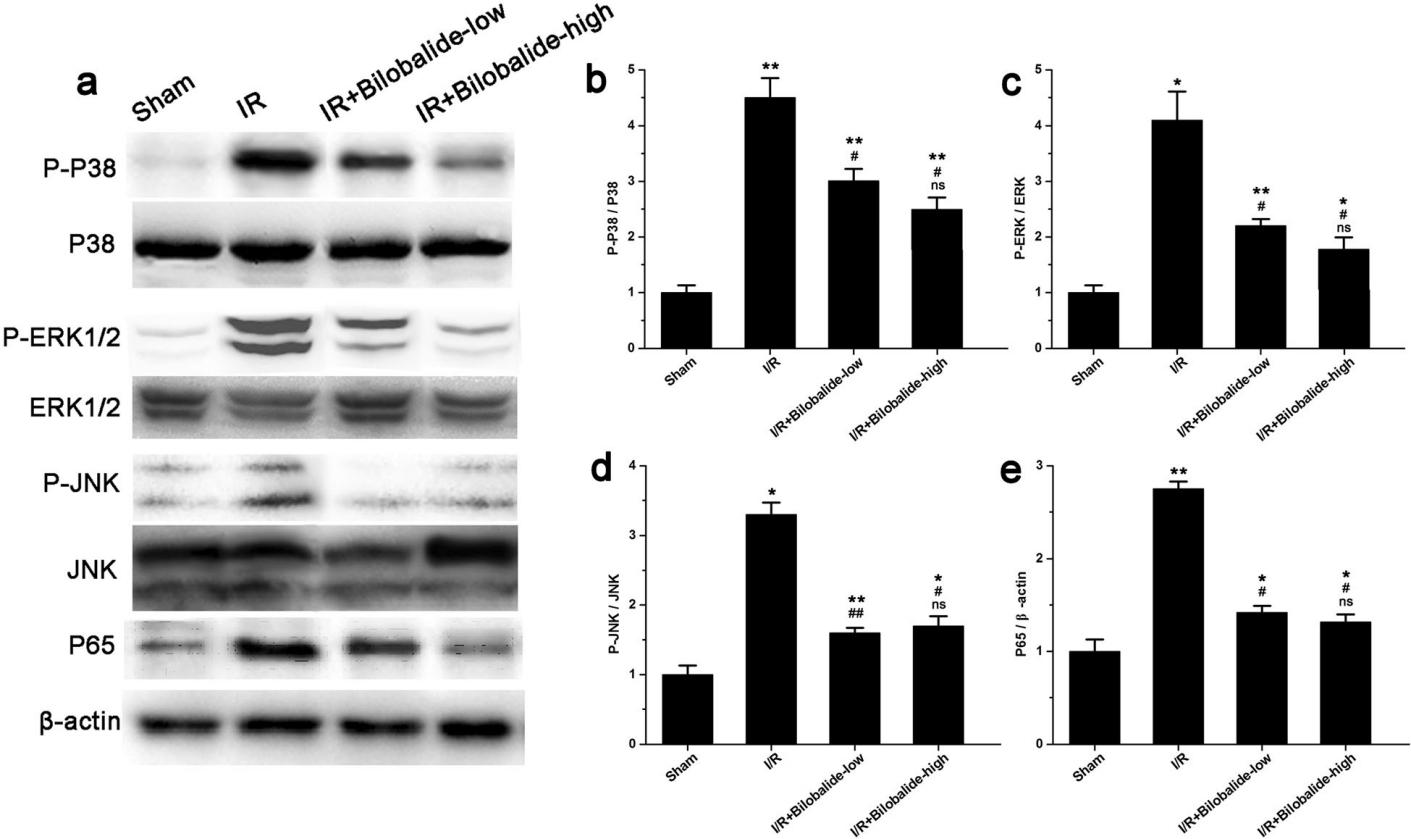
Bilobalide reduced the activation of p38, ERK1/2, JNK, and NF-휅B p65 in skeletal muscle following I/R. **a** Representative immunoblots of P-P38, total P38, P-ERK1/2, total ERK1/2, P-JNK, total JNK, P65 and β-actin in skeletal muscle. The total amount of protein loaded per lane was 40 μg. Detection of β-actin on the same blots was used to verify equal loading among the various lanes. **b**-**e** Bar graph illustrating the average relative expression of P-P38, total P38 (**b**), P-ERK1/2, total ERK1/2 (**c**), P-JNK, total JNK (**d**) and P65 (**e**) in each group. The protein levels were quantified by camera-based detection of emitted chemiluminescence. ^*^P < 0.05 and ^**^p < 0.01 compared with the Sham group; ^#^*p* < 0.05 and ^##^*p* < 0.01 compared with the I/R group; ns = not significant compared with the I/R + bilobalide-low group. *n* = 8 per group

As shown in Fig. 4, I/R injury alone increased the expression of phosphorylated p38 (Fig. 4b, *p* < 0.05), phosphorylated ERK1/2 (Fig. 4c, *p* < 0.05), phosphorylated JNK (Fig. 4d, *p* < 0.05) and NF- κB p65 (Fig. 4e, *p* < 0.05), while bilobalide administration significantly attenuated the I/R-induced upregulation of phosphorylated p38 (Fig. 4b, *p* < 0.05, vs. the I/R injury group), phosphorylated ERK1/2 (Fig. 4c, *p* < 0.05, vs. the I/R injury group), phosphorylated JNK (Fig. 4d, *p* < 0.05, vs. the I/R injury group) and NF- κB p65 (Fig. 4e, *p* < 0.05, vs. the I/R injury group). These findings suggest that the protective effects of bilobalide in this skeletal I/R injury model might be mediated partly through inhibition of p38, ERK1/2, JNK and NF- κB p65 pathway activation. However, the expression of phosphorylated p38 (Fig. 4b), phosphorylated ERK1/2 (Fig. 4c), phosphorylated JNK (Fig. 4d), and NF- κB p65 (Fig. 4e) was higher in the I/R + bilobalide-low group and the I/R + bilobalide-high group than in the sham group (*p* < 0.05). There was no significant difference in the expression of phosphorylated p38 (Fig. 4b), phosphorylated ERK1/2 (Fig. 4c), phosphorylated JNK (Fig. 4d), or NF- κB p65 (Fig. 4e) between the I/R + bilobalide-high group and the I/R + bilobalide-low group (*p* > 0.05).

## Discussion

In this study, we initially found that bilobalide partially restored the morphology of the gastrocnemius after I/R injury and reduced tissue edema in skeletal muscle. Furthermore, we demonstrated that bilobalide could relieve oxidative stress and systemic inflammatory responses caused by I/R injury. In addition, our findings suggested that bilobalide suppressed the activation of the p38, ERK1/2, JNK, and NF- κB p65 pathways in the I/R injured skeletal muscle, suggesting that the protective effects of bilobalide might be partly mediated by suppressing the activation of MAPK/NF- κB pathway.

Previous studies have indicated that skeletal muscle I/R injury is an important clinical problem that should not be ignored [1–3]. At present, there are different ways to treat limb I/R injury, including physical therapy and chemical therapy. It has been reported that ischemic preconditioning [9], ischemic postconditioning [21], controlled reperfusion [22], hypothermia [23], light-emitting diode therapy [24] and some other physical therapies can relieve skeletal I/R injury [25]. In addition, several agents, such as dexamethasone [10], curcumin [26], salvianolic acid [8], silibinin [27], simvastatin [28], cyclosporine A [29], hydrogen-rich saline [30] and lipoxin A4 [4], have been shown to be effective in attenuating skeletal I/R injury. In cases of traumatic injuries in which I/R is not predictable and early intervention is desired, such strategies are not as relevant. This is especially true for severe extremity injuries. Lifesaving surgical procedures are performed in these cases with the objective of stopping hemorrhage for the preservation of vital organ function; the extremities are not the primary focus. Moreover, even though the strategies and agents mentioned above have shown some benefits in the laboratory, none have been established to have any clinical benefits. Hence, there is still a need to discover novel substances with antioxidant and anti-inflammatory capacities that can be utilized for the treatment of skeletal I/R injury.

Bilobalide can be extracted from *Ginkgo biloba* leaves, which are widely used in traditional Chinese medicine. EGb 761, a standardized extract from *Ginkgo biloba* leaves, has various pharmacological functions and has been used worldwide. Bilobalide accounts for approximately 3% of EGb 761 [31]. Otani et al. reported that bilobalide can prevent cytotoxic brain edema caused by triethyltin [32]. Others have shown that bilobalide can protect against ischemia or I/R injury-induced edema formation and that it is a potential antiedema drug [33]. In the present study, we found that bilobalide attenuated I/R injury in a skeletal I/R injury model. Histological findings indicated that there were fewer histopathologic changes in bilobalide-treated animals subjected to I/R than in untreated animals subjected to I/R. Moreover, we demonstrated that bilobalide significantly reduced edema in I/R-injured skeletal muscle.

Oxidative stress is considered to play important roles in the process of limb I/R injury [8]. Our data showed that bilobalide significantly ameliorated skeletal muscle oxidative stress induced by I/R injury by decreasing NADPH oxidase activity and F_2_-isoprostane and MDA levels and by enhancing CAT, GSH-Px and SOD activity. Meanwhile, antioxidative effects of bilobalide have been reported in the context of neuronal degeneration in Alzheimer’s disease [34]. Parimoo et al. showed that bilobalide can be used as a promising hepatoprotectant due to its antioxidative effects [35]. Lu et al. demonstrated that bilobalide protects melanocytes from H_2_O_2_-induced oxidative damage by increasing antioxidant expression [36]. These results indicate that the antioxidative effects of bilobalide may be important in cases of I/R-injured skeletal muscle.

Inflammatory responses are generally considered to be major causes of skeletal muscle I/R injury [4]. Puntel and colleagues have illustrated that MPO activity is an indicator of neutrophil infiltration in I/R injury in skeletal muscle [37]. Infiltrating neutrophils can also release a variety of inflammatory cytokines, such as TNF-α, IL-1β and IL-6, which play roles in inflammatory reactions [38]. Several studies have demonstrated that bilobalide acts as an anti-inflammatory mediator in cerebral I/R injury and many other inflammatory response-related diseases [39–41]. In this study, we found that bilobalide could relieve systemic inflammatory responses in the skeletal muscle caused by I/R injury.

Under oxidative stress or inflammatory response conditions, the MAPK and NF- κB pathways can be activated [10]. Recent studies have shown that in some I/R injury models, including the skeletal I/R injury model, the p38, ERK1/2, JNK and p65 pathways are active [4, 8]. In the present study, we found that inhibition of these four specific cellular pathways with their specific inhibitors (P38 MAPK: SB203580; ERK: ravoxertinib; JNK: SP600125; P65 NF-κB: SC75741) alleviated apoptosis and local oxidative stress and decreased inflammatory cytokine levels in IR-injured skeletal muscle (Supplementary Figure 2). Additionally, we demonstrated that the protein levels of phosphorylated p38, phosphorylated ERK1/2, phosphorylated JNK, and p65 was significantly increased in skeletal muscle after I/R injury and that bilobalide partially inhibited p38, ERK1/2, JNK, and p65 activation, suggesting that activation of the corresponding signaling pathways might mediate some of the protective effects of bilobalide in this model. These observations are consistent with the findings of previous studies. Priyanka and colleagues showed that bilobalide abates inflammation induced by hypoxia in adipocytes via reducing activation of the NF- κB and JNK signaling pathways [40]. Zhou and colleagues showed that bilobalide inhibits the secretion of inflammatory factors in BV2 microglia in response to oxygen/glucose deprivation and reoxygenation by controlling the activation of TLR/MyD88/NF- κB pathways [41]. In other studies, however, bilobalide has failed to inhibit increases in p-ERK1/2 expression. For example, Jiang et al. proved that bilobalide could play an important role in neuroprotection against cerebral I/R injury through a mechanism related to downregulation of JNK and p38 activation but not ERK1/2 activation [42]. Previous studies have also revealed that bilobalide cannot regulate the expression of p-ERK1/2 in SH-SY5Y cells [43]. These variations in results may be associated with the different species and sources of cells or the different animal and disease models used.

There were some limitations to our study. In this study, we treated the rats with bilobalide after 3 h of ischemia and then reperfused them for 24 h. However, in some clinical cases (for example, during reattachment of a limb or flap), there is not enough time for bilobalide treatment between ischemic injury and reperfusion. We did not test whether administering bilobalide treatment after I/R injury had the same protective effects as administering it between ischemia and reperfusion. In addition, we observed involvement of the p38, ERK1/2, JNK and p65 pathways in the protective effects of bilobalide on skeletal muscle. However, the specific effects of these pathways in limb I/R injury remain to be investigated.

## Conclusions

In summary, our study reveals that bilobalide alleviates skeletal muscle damage caused by I/R injury. Moreover, bilobalide protects against I/R injury-induced oxidative stress and inflammation via the MAPK/NF- κB pathways. Bilobalide may be a promising drug for I/R-injured muscle tissue.

## Supplementary information

**Additional file 1: Supplementary Figure 1.** Bilobalide has no effects on healthy rats. (a) Representative photomicrographs of skeletal muscle tissue following H&E staining. (b) Edema is presented as the wet weight/dry weight ratio of skeletal muscle in the different groups. (c) Effects of bilobalide on local oxidative stress in skeletal muscle in rats with I/R injury. (d) Effects of bilobalide on the skeletal muscle inflammatory response in rats with I/R injury. ns = not significant. *n* = 3 per group. **Supplementary Figure 2.** Effects of inhibitors of MAPK and NF-κB pathways on IR-injured skeletal muscle. (a) Representative immunoblots of BAX, BCL-2 and β-actin in skeletal muscle (left). The total protein amount loaded per lane was 40 μg. Detection of β-actin on the same blots was used to verify equal loading among the various lanes. The bar graphs illustrate the average relative expression levels of BAX and BCL-2 in each group, which were quantified by camera-based detection of emitted chemiluminescence (middle and right). (b) Inhibitors of MAPK and NF-κB pathways reduced NADPH oxidase activity and enhanced SOD activity in skeletal muscle following I/R. (c) Inhibitors of MAPK and NF-κB pathways increased the levels of IL-1β and TNF-α in skeletal muscle following I/R. ^*^P < 0.05 compared with the sham group; ^#^*P* < 0.05 compared with the I/R group. *n* = 3 per group. **Supplementary Figure 3.** The uncropped Western blot scans.

## Data Availability

Authors declares that data and materials described in the manuscript are freely available to any scientist wishing to use them, without breaching participant confidentiality.

## Abbreviations

CAT: Catalase
GSH-Px: Glutathione peroxidase
H&E: Hematoxylin and eosin
IL-1β: Interleukin 1 beta
IL-6: Interleukin 6
I/R: Ischemia/reperfusion
MAPK: Mitogen-activated protein kinase
MDA: Malondialdehyde
MPO: Myeloperoxidase
NADPH: Nicotinamide adenine dinucleotide phosphate
NF-휅B: Nuclear factor kappa-B
ROS: Reactive oxygen species
SOD: Superoxide dismutase
TNF-α: Tumor necrosis factor alpha
