# Correction to: Bilobalide protects against ischemia/reperfusion-induced oxidative stress and inflammatory responses via the MAPK/NF-κB pathways in rats

**DOI:** 10.1186/s12891-020-03607-5

**Published:** 2020-08-31

**Authors:** Ying Li, Jiliang Jiang, Liangcheng Tong, Tingting Gao, Lei Bai, Qing Xue, Jianxin Xing, Qin Wang, Haoran Lyu, Min Cai, Zhongyang Sun

**Affiliations:** 1 Department of Orthopedics, Air Force Hospital of Eastern Theater, Anhui Medical University, Nanjing, China; 2 Department of Neurosurgery, Yulin First Hospital, the Second Affiliated Hospital of Yan’an University, Yulin, China; 3 Department of Orthopedics, Yuhuatai Hospital, Nanjing, China; 4 Department of Orthopedics, Zhangwenxin Hospital, Nanjing, China

**Correction to: BMC Musculoskelet Disord 21, 449 (2020)**

**https://doi.org/10.1186/s12891-020-03479-9**

Following publication of the original article [1], the authors noticed that the article title has a garbled code.

The correct title is shown below.

“Bilobalide protects against ischemia/reperfusion-induced oxidative stress and inflammatory responses via the MAPK/NF-κB pathways in rats”

The original article [1] has been updated.
