# Supplementary material for: Bilobalide protects against ischemia/reperfusion-induced oxidative stress and inflammatory responses via the MAPK/NF-휅B pathways in rats
Source: BMC Musculoskelet Disord. 2020 Jul 9;21:449. doi: 10.1186/s12891-020-03479-9 (PMC7350583; doi:10.1186/s12891-020-03479-9)
Supplement: Supplementary file 1 — Additional file 1: Supplementary Figure 1. Bilobalide has no effects on healthy rats. (a) Representative photomicrographs of skeletal muscle tissue following H&E staining. (b) Edema is presented as the wet weight/dry weight ratio of skeletal muscle in the different groups. (c) Effects of bilobalide on local oxidative stress in skeletal muscle in rats with I/R injury. (d) Effects of bilobalide on the skeletal muscle inflammatory response in rats with I/R injury. ns = not significant. n = 3 per group. Supplementary Figure 2. Effects of inhibitors of MAPK and NF-κB pathways on IR-injured skeletal muscle. (a) Representative immunoblots of BAX, BCL-2 and β-actin in skeletal muscle (left). The total protein amount loaded per lane was 40 μg. Detection of β-actin on the same blots was used to verify equal loading among the various lanes. The bar graphs illustrate the average relative expression levels of BAX and BCL-2 in each group, which were quantified by camera-based detection of emitted chemiluminescence (middle and right). (b) Inhibitors of MAPK and NF-κB pathways reduced NADPH oxidase activity and enhanced SOD activity in skeletal muscle following I/R. (c) Inhibitors of MAPK and NF-κB pathways increased the levels of IL-1β and TNF-α in skeletal muscle following I/R. *P < 0.05 compared with the sham group; #P < 0.05 compared with the I/R group. n = 3 per group. Supplementary Figure 3. The uncropped Western blot scans. [file 12891_2020_3479_MOESM1_ESM.docx]

**Bilobalide protects against ischemia/reperfusion-induced oxidative stress and inflammatory responses via the MAPK/NF-𝜅B pathways in rats**

Ying Li ^a^, Jiliang Jiang ^a^, Liangcheng Tong ^a^, Tingting Gao ^a^, Lei Bai ^b^, Qing Xue ^a^, Jianxin Xing ^c^, Qin Wang ^d^, Haoran Lyu ^a^, Min Cai ^a^, Zhongyang Sun ^a,*^

^a^ Department of Orthopedics, Air Force Hospital of Eastern Theater, Anhui Medical University, Nanjing, China.

^b^ Department of Neurosurgery, Yulin First Hospital, the Second Affiliated Hospital of Yan’an University, Yulin, China.

^c^ Department of Orthopedics, Yuhuatai Hospital, Nanjing, China.

^d^ Department of Orthopedics, Zhangwenxin Hospital, Nanjing, China.


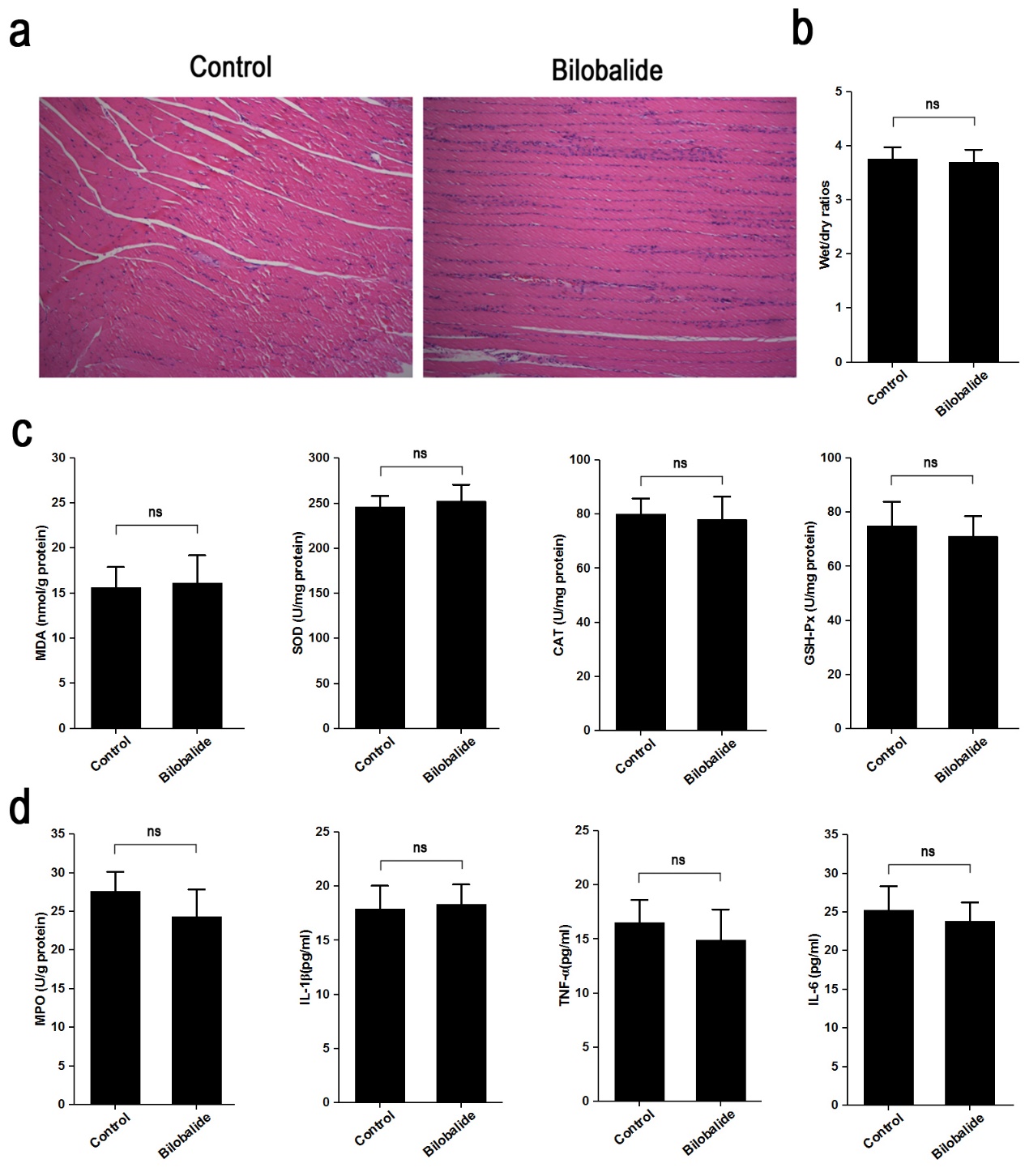


**Supplementary Figure 1.** Bilobalide has no effects on healthy rats. (a) Representative photomicrographs of skeletal muscle tissue following H&E staining. (b) Edema is presented as the wet weight/dry weight ratio of skeletal muscle in the different groups. (c) Effects of bilobalide on local oxidative stress in skeletal muscle in rats with I/R injury. (d) Effects of bilobalide on the skeletal muscle inflammatory response in rats with I/R injury. ns=not significant. 𝑛=3 per group.


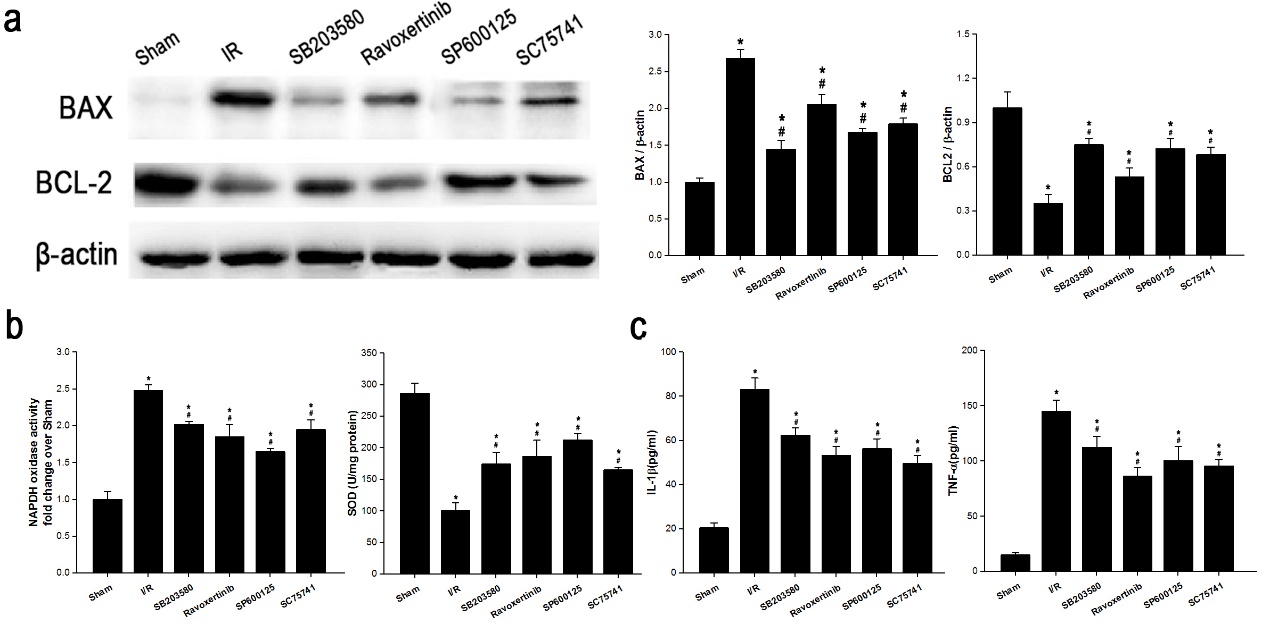


**Supplementary Figure 2. Effects of inhibitors of MAPK and NF-κB pathways on IR-injured skeletal muscle.** (a) Representative immunoblots of BAX, BCL-2 and β-actin in skeletal muscle (left). The total protein amount loaded per lane was 40 μg. Detection of β-actin on the same blots was used to verify equal loading among the various lanes. The bar graphs illustrate the average relative expression levels of BAX and BCL-2 in each group, which were quantified by camera-based detection of emitted chemiluminescence (middle and right). (b) Inhibitors of MAPK and NF-κB pathways reduced NADPH oxidase activity and enhanced SOD activity in skeletal muscle following I/R. (c) Inhibitors of MAPK and NF-κB pathways increased the levels of IL-1β and TNF-α in skeletal muscle following I/R. ^*^*P*<0.05 compared with the sham group; ^#^*P*<0.05 compared with the I/R group. 𝑛=3 per group.

**
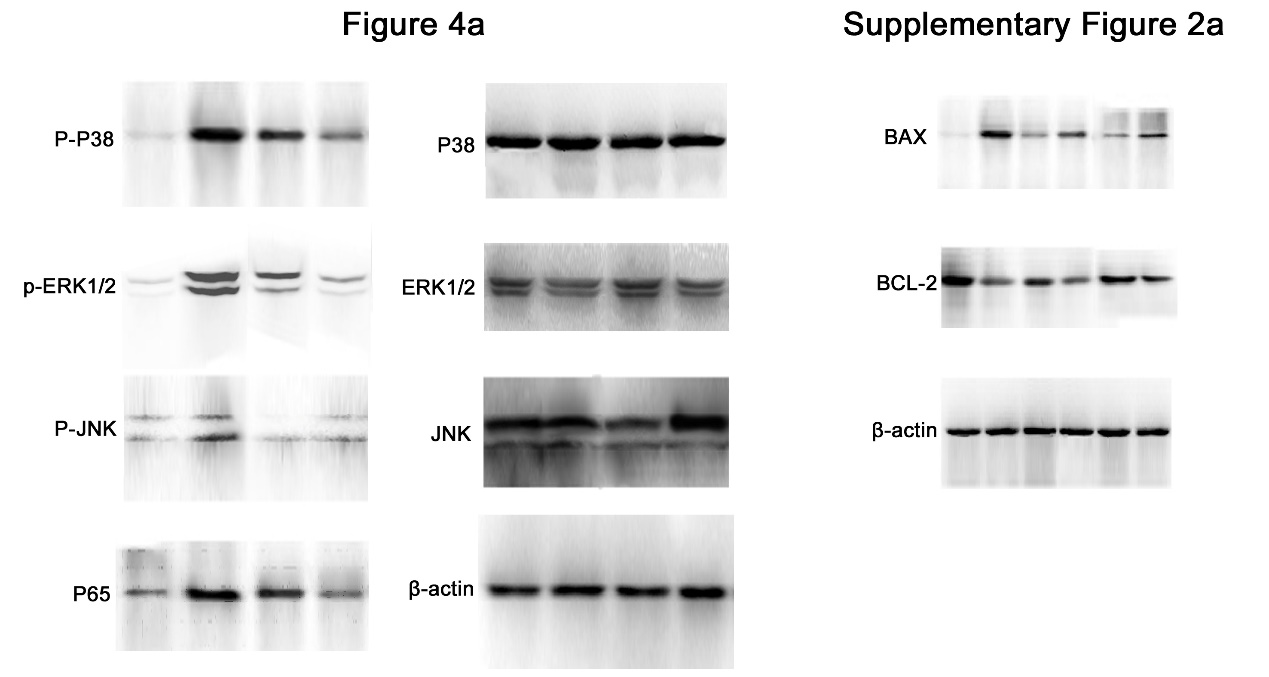
Supplementary Figure 3. The uncropped Western blot scans.**
